# An Algae Cultivator Coupled with a Hybrid Photosynthetic–Air-Cathode Microbial Fuel Cell with Ceramic Membrane Interface

**DOI:** 10.3390/membranes15100295

**Published:** 2025-09-30

**Authors:** Chikashi Sato, Ghazaleh Alikaram, Oluwafemi Oladipupo Kolajo, John Dudgeon, Rebecca Hazard, Wilgince Apollon, Sathish-Kumar Kamaraj

**Affiliations:** 1 Department of Civil and Environmental Engineering, Idaho State University, 921 S. 8th Ave., Stop 8060, Pocatello, ID 83209, USA; ghazalehalikaram@isu.edu (G.A.); kolajooluwafemi@isu.edu (O.O.K.); 2 Center for Archaeology, Materials and Applied Spectroscopy (CAMAS), Idaho State University, 921 South 8th Avenue, Stop 8094, Pocatello, ID 83209, USA; johndudgeon@isu.edu (J.D.); rebeccahazard@isu.edu (R.H.); 3 Centro de Investigación en Ciencia Aplicada y Tecnología Avanzada (CICATA), Instituto Politécnico Nacional (IPN), Carretera Tampico-Puerto Industrial Altamira km 14.5, C. Manzano, Industrial Altamira, Altamira 89600, Mexico; wilgince.apollon@uanl.edu.mx (W.A.); skamaraj@ipn.mx (S.-K.K.)

**Keywords:** ceramic membrane, *Chlorella vulgaris*, diffusion, mass flux, microbial fuel cell, microalgae, nutrient recovery, wastewater treatment

## Abstract

Microalgae are promising candidates for renewable biofuel production and nutrient-rich animal feed. Cultivating microalgae using wastewater can lower production costs but often results in biomass contamination and increases downstream processing requirements. This study presents a novel system that integrates an algae cultivator (AC) with a single-chamber microbial fuel cell (MFC) equipped with photosynthetic and air-cathode functionalities, separated by a ceramic membrane. The system enables the generation of electricity and production of clean microalgae biomass concurrently, in both light and dark conditions, utilizing wastewater as a nutrient source and renewable energy. The MFC chamber was filled with simulated potato processing wastewater, while the AC chamber contained microalgae *Chlorella vulgaris* in a growth medium. The ceramic membrane allowed nutrient diffusion while preventing direct contact between algae and wastewater. This design effectively supported algal growth and produced uncontaminated, harvestable biomass. At the same time, larger particulates and undesirable substances were retained in the MFC. The system can be operated with synergy between the MFC and AC systems, reducing operational and pretreatment costs. Overall, this hybrid design highlights a sustainable pathway for integrating electricity generation, nutrient recovery, and algae-based biofuel feedstock production, with improved economic feasibility due to high-quality biomass cultivation and the ability to operate continuously under variable lighting conditions.

## 1. Introduction

### 1.1. Background

The intertwined global challenges of population growth, rapid industrialization, and environmental degradation are driving unprecedented demand for renewable energy, clean water, and sustainable food production [1]. Meanwhile, growing volumes of wastewater demand effective treatment and reuse, underscoring the need for integrated solutions that address multiple needs while maintaining both environmental and economic sustainability. A secure energy supply is fundamental for global economic stability. Yet, as of 2024, fossil fuels remain the dominant energy source, accounting for approximately 87% of global energy demand and emitting an estimated 40.8 gigatons of CO_2_ annually [2]. These emissions intensify the greenhouse effect and accelerate climate change, underscoring the urgent need for renewable energy alternatives with minimal carbon footprints. While solar, wind, and hydropower have made substantial contributions toward reducing fossil fuel dependence [2], biofuels offer a unique advantage: they can serve both as renewable energy sources and as chemical energy storage [3]. Among these, microalgae-based biofuels are particularly attractive due to their high productivity, ability to capture CO_2_, and compatibility with wastewater utilization.

### 1.2. Microalgae

Algae-based biofuels, classified as third-generation biofuels, are promising due to their rapid growth rates, high biomass productivity, and efficient assimilation of nutrients such as phosphorus (P), nitrogen (N), and CO_2_ [4,5]. Microalgae, in particular, offer a low-carbon-footprint feedstock for biofuel production and can also be used as nutrient-rich livestock feed [6,7,8] and fish feed [8,9,10]. Their cultivation does not compete with food crops for arable land [5,11], and many species are rich in lipids and polysaccharides for biodiesel, as well as proteins, vitamins, and pigments of commercial value [7,12,13]. Microalgae lack lignin, which simplifies downstream processing compared to lignocellulosic biomass. Under controlled conditions, their growth can be predicted [14], and many species thrive in fresh or saltwater, enabling near year-round harvesting [6,15]. However, large-scale production remains economically challenging due to high harvesting, pretreatment, and processing costs [16]. One promising strategy to reduce these costs is to use nutrient-rich wastewater for cultivation. Microalgae can utilize agricultural, domestic, and industrial wastewater as nutrient sources [17]. Yet, direct exposure of algal biomass to wastewater risks contamination, requiring extensive preprocessing before downstream use. In this study, an algae cultivator (AC) is integrated with a microbial fuel cell (MFC) to enable nutrient extraction from wastewater without direct algae–wastewater contact. This approach aims to remove organic matter and nutrients from wastewater, generate electricity, and simultaneously grow uncontaminated algal biomass.

### 1.3. Microbial Fuel Cells (MFCs)

Microbial fuel cells (MFCs) are bioelectrochemical systems that convert the chemical energy stored in organic compounds directly into electricity using electrogenic or electroactive bacteria (EAB) as biocatalysts [18,19]. MFCs are of interest because they offer simultaneous renewable electricity generation and wastewater treatment. Structurally, MFCs are commonly classified into dual-chamber and single-chamber configurations. In a dual-chamber MFC, the anode and cathode are separated by a proton exchange membrane (PEM). Within the anode chamber, EAB oxidize organic substrates, releasing protons (H^+^), electrons (e^−^), CO_2_, and residual by-products. Protons migrate through the PEM to the cathode chamber, while electrons flow via an external circuit to the cathode, where they reduce an electron acceptor, often oxygen, thus completing the circuit. In single-chamber MFCs, the cathode chamber is eliminated, and the cathode is directly exposed to atmospheric oxygen, simplifying the design and reducing costs. The present study used a single-chamber air-cathode MFC.

### 1.4. Photosynthetic MFCs (pMFCs)

Photosynthetic MFCs (pMFCs) integrate MFC technology with algae cultivation, using oxygen produced during algal photosynthesis as the cathodic electron acceptor [20]. This eliminates the need for external aeration, lowering energy demands while enabling simultaneous power generation, nutrient recovery, and biomass production. As is summarized in Table A1 (Appendix A), numerous pMFC configurations have been explored, including sequential anode–cathode arrangements [21], two-chamber systems coupled with algal bioreactors [22], tubular designs fed with digested kitchen waste [23], and top–bottom dual chambers treating dairy wastewater [24]. These examples demonstrate the versatility of pMFCs; however, scalable deployment is hindered by challenges in cost-effective algal cultivation and downstream processing.

### 1.5. Ceramic Membrane-Based MFCs

Synthetic polymeric ion exchange membranes such as Nafion-117, made from perfluorosulfonic acid, are widely used in MFC research for their excellent proton conductivity. However, their high cost often represents a major portion of total MFC expenses, making large-scale applications economically prohibitive [25,26,27,28]. Ceramic membranes offer a low-cost, durable alternative with favorable electrochemical and physical properties [25,27,29,30,31,32]. They possess high mechanical strength, chemical stability, thermal resistance, bacterial resistance, and ion permeability. Furthermore, ceramics are made from abundant, low-cost raw material, clay, which is environmentally benign and easily disposed of after use [28,29]. Variations in ceramic membrane-based MFCs and their performance are summarized in Table A2 (Appendix B). In some cases, ceramic membranes can perform comparably, or even better than Nafion. For example, Das et al. [33] reported slightly higher power density in a ceramic membrane MFC containing goethite compared to one with Nafion-117, at roughly one-fifth the cost. In the present study, the ceramic membrane serves as both the MFC separator and the nutrient-transfer interface to the algae cultivator.

### 1.6. Goal and Objective

The goal of this study is to reduce the overall cost of microalgae biofuel production by integrating the complementary functions of a microbial fuel cell and an algae cultivator. The specific objectives are to develop and evaluate a Microbial Fuel Cell–Algae Cultivator (MFC–AC) system that:

- Produces clean algal biomass for biofuel without direct contact between algae and wastewater, while recovering nutrients from the wastewater.
- Recovers carbon from wastewater as algal biomass, thereby reducing CO_2_ emissions to the atmosphere.
- Recovers electrical energy from wastewater while removing organic matter and nutrients.

## 2. Materials and Methods

### 2.1. Wastewater (Substrate) and Bacterial Inoculum

To maintain consistency and reproducibility, a simulated potato-process wastewater was used as the substrate (anolyte) for the MFC. Actual industrial wastewater often varies in composition and concentration, which can complicate performance comparisons. The simulated wastewater was prepared by diluting 10 mL (12.7 g) of concentrated potato extract and 200 mL of 1 M phosphate buffer (pH 7) in 2000 mL of deionized (DI) water. The mixture was sterilized by autoclaving at 121 °C for 20 min and stored at 4 °C until use. This solution served both as the fuel source for electricity generation and as a growth medium for the anode microbial community. The concentrated potato extract was obtained from a local food processing facility in Idaho, USA. The chemical characteristics of the simulated wastewater are summarized in Table 1.

An anaerobic mixed microbial consortium served as the biocatalyst for the MFC anode. The culture was sourced from sludge collected at an anaerobic digester in a municipal wastewater treatment facility in Pocatello, Idaho, USA, and has been maintained for many years in our laboratory using simulated potato-process wastewater in MFC reactors. The culture has been used in previous studies [1,34,35,36]. Microbial community analyses have shown *Proteobacteria*, *Firmicutes*, and *Bacteroidetes* as the dominant phyla [34].

### 2.2. Microalgae

A pure culture of *Chlorella vulgaris* (Algae Research and Supply, Carlsbad, CA, USA) was cultivated in OECD medium following Adochite and Andronic [37]. Cultures were grown in 75 cm^2^ vent-cap flasks (Celltreat Scientific Products, Ayer, MA, USA) at 22 ± 1 °C. Algal concentrations were quantified by developing a standard curve relating biomass to optical density at 600 nm using a UV–Vis spectrophotometer (Cary 3500, Agilent, Palo Alto, CA, USA). Dry mass was determined gravimetrically by filtering, drying, and weighing samples, then dividing by sample volume to obtain g L^−1^. Once the correlation was established, routine biomass measurements were performed spectrophotometrically.

### 2.3. MFC Design and Operation

Two single-chamber, air-cathode MFCs (MFC 1 and MFC 2) were built from acrylic sheets following the design in Sato et al. [35]. Each chamber (13 × 9 × 11 cm) had sealed inlet/outlet ports to maintain anaerobic conditions. One side contained a 3 cm-diameter opening covered by a ceramic membrane and a cathode. The cathode electrode was made of carbon cloth with a platinum loading of 0.3 mg cm^−2^ (Fuel Cell Earth, Woburn, MA, USA) and had a geometric surface area (one side) of 6.7 cm^2^. The anode consisted of bamboo charcoal (BC) plates (Mt Meru Pte, Singapore). Four BC plates were positioned 2 cm apart, with the nearest plate located 3 cm from the cathode. Each BC plate had a geometric surface area of approximately 75 cm^2^ (range: 64–81 cm^2^) and an average electrical resistance of 30 Ω (range: 16–54 Ω) measured in both lengthwise and widthwise directions. The surface morphology of the BC plates was examined using a scanning electron microscope (SEM) (Quanta 200 FEG, FEI Co., Brno, Czech Republic). Stainless steel wire leads were attached to each electrode using conductive epoxy adhesive (MG Chemicals, Burlington, ON, Canada) and subsequently reinforced and insulated with nonconductive epoxy putty (J-B Weld, Coopersburg, PA, USA). The anode and cathode were connected through an external resistor, 974 Ω for MFC 1 and 977 Ω for MFC 2, to complete the electrical circuit. The installation of the BC anode plates resulted in a working (anolyte) volume of approximately 500 mL for the MFC. Ceramic membranes (0.4 cm thick) were fabricated from clay using the method of Paucar and Sato [36] and characterized in Sato et al. [1].

### 2.4. Algae Cultivator (AC) Design and Operation

The AC was a transparent plastic vessel (20 × 33 × 11.5 cm) with a rectangular lid opening (9.5 × 13.5 cm) to fit the MFC chamber. This allowed direct contact between the MFC cathode and the algal medium. The vessel was covered with a thin transparent sheet to reduce evaporation while transmitting light for photosynthesis. During the operation, DI water was periodically added to maintain ~2.5 L working volume. Illumination was provided by 5500 K daylight grow lights (GHODEC, Shenzhen, China) on a 12 h light/dark cycle. Photosynthetically active radiation (400–700 nm) averaged 64.85 ± 5.27 µmol m^−2^ s^−1^ (*n* = 24), measured with a PAR meter (QPM-200, Quantum Sun, Vancouver, BC, Canada).

### 2.5. MFC–AC System Operation

**Figure 1:**
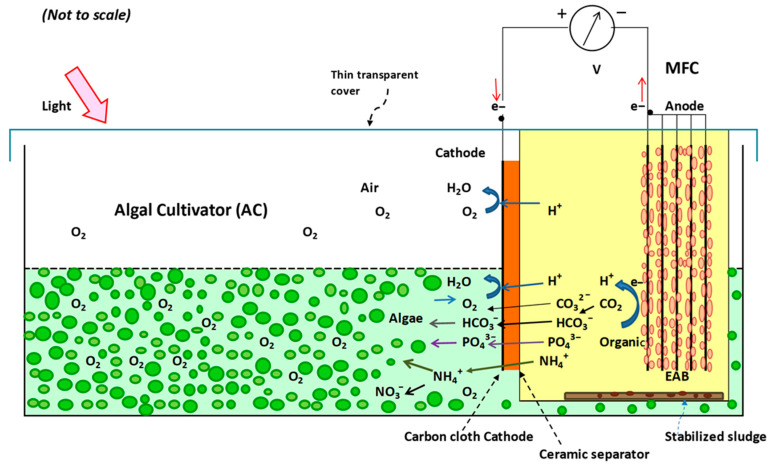
Conceptual model of MFC-AC system.

The single-chamber air-cathode MFC was integrated into the AC (Figure 1) with half of the cathode area submerged in water. This enabled dual operation as an air-cathode MFC and a photosynthetic MFC (pMFC) under both light and dark conditions. Before integration, MFCs were operated separately until a stable voltage ≥ 0.1 V was achieved (~2 weeks). They were then emptied, refilled with fresh substrate, and placed in the AC. Two independent systems (MFC-AC 1 and MFC-AC 2) operated in batch mode at 22 ± 1 °C for 42 days.

### 2.6. Water and Wastewater Analyses

Water samples from the AC vessels were collected at ~7-day intervals, while wastewater samples from the MFCs were taken only at the beginning and end of each run to preserve anaerobic conditions. Before sampling, the AC vessel water was homogenized using a magnetic stirrer (Nuova II, Thermolyne, Waltham, MA, USA).

Samples from both AC vessels and MFCs were analyzed for key water quality parameters, including pH, conductivity, alkalinity, chemical oxygen demand (COD), ammonium-nitrogen (NH_4_^+^-N), nitrate-nitrogen (NO_3_^−^-N), phosphate-phosphorus (PO_4_^3−^-P), and selected inorganic elements. All samples, except those for pH, alkalinity, and conductivity, were filtered through a 0.45 μm syringe filter (Cobetter, Hangzhou, China). pH and conductivity were measured using a pH meter (LLC-AI501 pH700, Apera Instruments, Columbus, OH, USA) and a conductivity meter (HI8733, Hanna Instruments, RI, USA), respectively. Alkalinity was determined by potentiometric titration with an autotitrator (Orion Star T900, Thermo Scientific, Waltham, MA, USA). COD was analyzed using a modified EPA Standard Method 5220 D with HACH digestion vials (HACH Company, Loveland, CO, USA). Phosphate (PO_4_^3−^) was determined using a modified Chen et al. [38] method, ammonium (NH_4_^+^) using modified procedures from Holmes et al. [39] and Solórzano [40], and nitrate (NO_3_^−^) using EPA Method 352.1. Elemental concentrations (e.g., Na, K, P) were measured by inductively coupled plasma mass spectrometry (ICP-MS; X-Series II, Thermo Scientific, Bremen, Germany). All reagents were of analytical grade. Unless otherwise specified, analyses were conducted in triplicate. Data were processed in Microsoft Excel (version 16) and reported as mean ± standard error. Additionally, ion speciation and saturation indices (SI) of AC water were calculated using Visual MINTEQ v3.1 [41].

### 2.7. Ceramics Analysis

The clay used in the construction of the ceramic separators for this project was analyzed by a combination of scanning electron microscopy (FEI Quanta FEG 200, Brno, Czech Republic) and energy-dispersive X-ray spectroscopy (Bruker QUANTAX 200, Billerica, MA, USA), SEM/EDS, to visualize the fired clay fabric and estimate the size and distribution of pores, and laser ablation ICP-MS (LA-ICP-MS), to determine the trace elements present in the clay matrix. Powder X-ray Diffraction (PXRD; Bruker D2 Phaser, Madison, WI, USA) and SEM/EDS on native, natural Peruvian clay (no added temper or other amendments) were performed in the CAMAS lab at Idaho State University.

### 2.8. Electrical Power Output Measurements

Voltage output (V, in volts) was continuously recorded at 15 min intervals using a data acquisition board (PCI-6024E) connected to a terminal block (SCB-68) and operated through LabVIEW (version 15) software [42]. Current (I, in amperes) was calculated using Ohm’s law: I = V/R_load_, where R_load_ is the external resistance (Ω). Power (P, in watts) was determined as P = I·V. Power density (PD) and current density (CD) were normalized to the cathode surface area (A_a_ = 6.7 cm^2^) according to PD = P/A_a_ (mW m^−2^) and CD = I/A_a_ (mA m^−2^), respectively. Polarization curves were generated by varying external resistance from 2000 to 20 Ω. Internal resistance was estimated based on the maximum power transfer theorem [43].

## 3. Results and Discussion

### 3.1. Bamboo Charcoal Electrode

Electrodes are critical components of MFCs, serving as the sites where electron transfer reactions occur [44]. Intending to scale up MFC systems, considerable research has focused on developing electrodes that enhance performance while reducing manufacturing costs [45]. The anode, in particular, plays a pivotal role, as its performance is directly influenced by extracellular electron transport at the interface between the anode surface and the surrounding anolyte [46]. Biochar has emerged as a promising low-carbon-footprint, renewable, and environmentally friendly alternative for bioelectrochemical processes. Its desirable characteristics include high surface area, porous structure, strong biocompatibility, chemical stability, and reasonable electrical conductivity [3].

**Figure 2:**
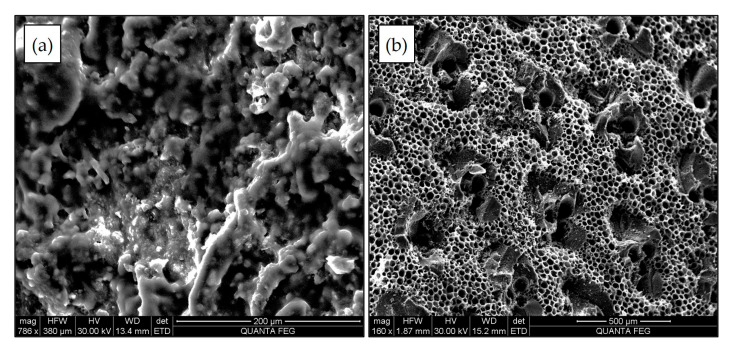
SEM images of bamboo charcoal anode: (**a**) external surface; (**b**) cross-section.

In this study, the bamboo charcoal (BC) used as the anode material exhibited a rough exterior with dense, round-to-hexagonal tubular structures, 20–500 µm in diameter, arranged in a honeycomb-like pattern (Figure 2). This porous architecture offers extensive surface area for microbial adhesion and biofilm development, while also promoting nutrient and proton transport, factors that are essential for high-performance anodes. However, one inherent drawback of BC is the natural variability in its physical and electrical properties, such as thickness and resistance [1]. Previous comparative studies have highlighted the potential of BC as an anode material. Moqsud et al. [47] reported that BC performed effectively when compared to carbon fiber anodes. Zhang et al. [48] further showed that tubular BC outperformed tubular graphite in terms of surface roughness, biocompatibility, electron transfer capacity, and internal resistance. Beyond electrochemical performance, bamboo offers significant environmental and economic benefits. It is a fast-growing plant [49] that rapidly sequesters atmospheric CO_2_ and stabilizes it in solid form [50]. Additionally, BC can be produced at low cost [51], reused, and safely disposed of (e.g., as biochar), reinforcing its value as a sustainable electrode material.

### 3.2. Characterization of Ceramic Membrane Separator

**Figure 3:**
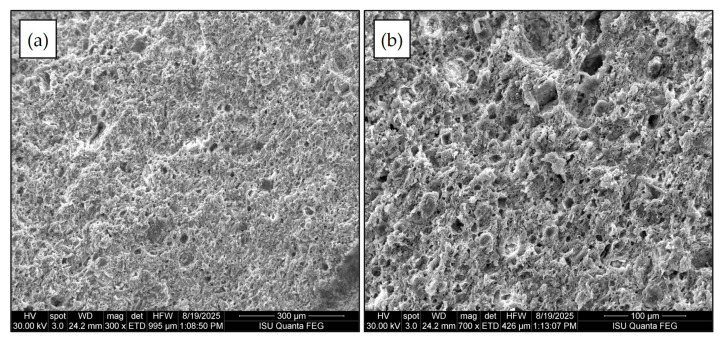
SEM images of fired ceramic membrane surfaces: (**a**) magnification of 300×, Horizontal Field Width (HFW) of 995 µm; (**b**) magnification of 700×, HFW 426.

In this study, ceramic membranes were fabricated from Peruvian clay following the method described by Paucar and Sato [36]. Representative regions of the membrane selected for microscopic imaging are shown in Figure 3. The Peruvian clay ceramic membrane exhibits a well-sorted matrix with fewer coarse grains compared to typical craft ceramics. Its surface displays a uniform distribution of pores, predominantly in the 10–20 μm diameter range, indicating a more homogeneous pore structure across the fired clay fabric.

The estimation of major and minor elements based on multiple area measurements on natural Peruvian clay using SEM/EDS is presented in Table 2. The trace elements in the clay matrix determined using the laser ablation ICP-MS (LA-ICP-MS) are presented in Table 3. The elemental analysis of the separator membranes shows that the clays are predominantly aluminum phyllosilicate, with variable concentrations of major and minor elements (Table 2).

**Figure 4:**
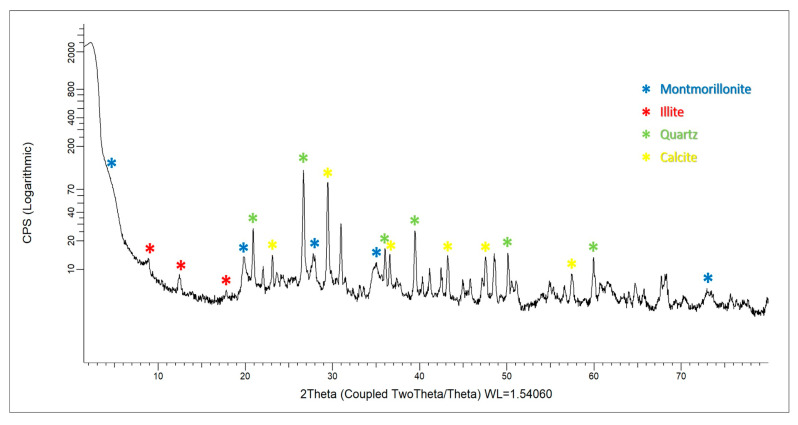
Powder X-ray diffraction (PXRD) analysis of the raw clay used for the membrane.

A powder X-ray diffraction analysis was performed on the raw Peruvian clays to determine their clay species (Figure 4). Raw clay pellets were ground in a mortar and pestle and sieved to collect the <62 µm (U.S. standard mesh 230) fraction. The 2*θ* region of analysis was 5–80, with a 0.5 s^−1^/step. Characteristic peaks were identified for at least two clays (illite and montmorillonite), as well as the presence of quartz and calcite.

Our analysis suggests a possible natural illite/montmorillonite mixture, based on observed potassium concentrations and magnesium–aluminum substitution in the clay. A silt fraction (2–50 µm) was observed with SEM/EDS in the clay, containing quartz, calcite, and feldspar inclusions. These inclusions act as a natural tempering agent in the clay during 650 °C firing, yielding a strong and durable ceramic. Montmorillonite clay is noted for its high workability for pottery production and its high cation exchange potential. It has many industrial and pharmacological uses [52,53] and has shown potential as a sequestration medium for heavy metals [51,54,55]. Montmorillonite clays are common in Peru [56] and are associated with weathering products from the Andes Mountain range.

The ceramic membrane separators used in MFC-AC 1 and MFC-AC 2 were further evaluated by determining their solute mass flux rate, F_x_. For this test, the algal culture vessels were filled with DI water without algae, while the MFC chambers contained simulated potato-process wastewater inoculated with the microbial consortium described in Section 2. Sodium (Na^+^) was selected as the model solute because it is chemically stable, highly mobile in water, and abundant in the wastewater. Consequently, salinity can serve as a reliable tracer for estimating transport parameters such as the dispersion coefficient [57].

The solute mass flux rate was calculated using Equation (1):

F_x_ = (dC/dt)V_v_/A_x_ (1)

where F_x_ = solute mass flux rate (g m^−2^ d^−1^), dC/dt = slope of the concentration–time plot (g m^−3^ d^−1^), V_v_ = AC vessel volume (m^3^), and A_x_ = ceramic membrane interface area (m^2^). During the initial phase of operation, Na^+^ concentrations in the AC vessels increased linearly over time. The slopes (dC/dt) were 0.568 mg L^−1^ d^−1^ (R^2^ = 0.901) for MFC-AC 1 and 0.422 mg L^−1^ d^−1^ (R^2^ = 0.917) for MFC-AC 2, which correspond to 0.568 g m^−3^ d^−1^ and 0.422 g m^−3^ d^−1^, respectively. Given an AC volume (V_v_) of 2.5 × 10^−3^ m^3^ and a membrane interface area (A_x_) of 3.35 × 10^−4^ m^2^, the calculated F_x_ values were 0.424 g m^−2^ d^−1^ for MFC-AC 1, and 0.315 g m^−2^ d^−1^ for MFC-AC 2. The higher Na^+^ flux in MFC-AC 1 may reflect greater membrane porosity or structural differences that promote solute diffusion. This enhanced transport could improve nutrient availability in the algal chamber, potentially contributing to higher system performance.

### 3.3. Voltage Output

**Figure 5:**
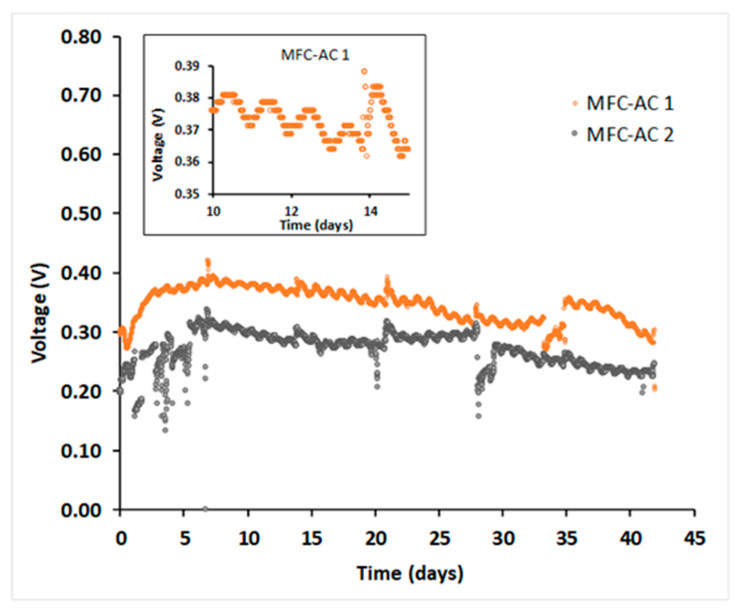
Voltage produced by MFC-AC 1 and MFC-AC 2.

The MFC-AC systems, inoculated with *C. vulgaris* in OECD medium, were operated under a 12 h light/dark cycle with a photosynthetically active radiation (PAR) of 64.85 ± 5.27 µmol m^−2^ s^−1^ (*n* = 24). Voltage was recorded every 15 min for six weeks (Figure 5). During the first week, MFC-AC 2 showed notable disturbances, likely reflecting system stabilization. Both systems exhibited periodic voltage fluctuations during water sampling and polarization tests.

On average, MFC-AC 1 generated a higher voltage (0.346 ± 0.001 V) than MFC-AC 2 (0.270 ± 0.001 V), suggesting higher bioelectrochemical activity and possibly more efficient nutrient transport. When compared with similar pMFC systems, MFC-AC 1 demonstrates competitive performance. For example, Kakarla and Min [58] reported 0.47 ± 0.03 V in an H-type pMFC with a Nafion-117 membrane, while Uggetti and Puigagut [59] achieved only 16 mV in a membrane-less pMFC treating filtered primary effluent with mixed algae. Khandelwal and Lens [24] obtained a peak of 0.125 V in a top–bottom two-chamber pMFC at 400 mg L^−1^ sulfide, and Wang et al. [60] observed ~0.412 V in a cylindrical two-chamber pMFC using synthetic biogas slurry. Ullah and Zeshan [61] reported 0.414 V in a two-chamber system treating untreated domestic wastewater. Recently, Zieliński et al. [62] reported ~0.42 V in an H-type two-chamber pMFC with a Nafion-117 membrane separator fed with dairy wastewater. These comparisons highlight that our MFC-AC 1 not only achieves voltage outputs on par with or exceeding many reported systems, but also maintains separation of algae and wastewater, demonstrating its potential for simultaneous electricity generation and clean algae cultivation.

### 3.4. Impact of Light and Dark Cycles

Light availability is a key factor influencing pMFC performance, as algal photosynthesis depends on both light intensity and wavelength. The inset of Figure 5 shows the voltage output from MFC-AC 1 between Days 10 and 15, illustrating the effect of the light/dark cycle. During the light phase, increased algal photosynthesis likely elevated dissolved oxygen (O_2_) levels in the AC vessel, leading to higher voltage output. Conversely, during the dark phase, reduced photosynthetic activity lowered O_2_ levels, causing a slight voltage drop. The measured fluctuation was ~0.02 V (~6% lower in the dark). For comparison, Zieliński et al. [62] reported much larger diurnal variations, ranging from ~0.42 V (in light) to ~0.2 V (in dark) with *Arthrospira platensis*, and from ~0.38 V (in light) to ~0.08 V (in dark) with *Chlorella vulgaris* in an H-type two-chamber pMFC fed with dairy wastewater. Similarly, Ullah and Zeshan [61] reported large diurnal variations in a two-chamber pMFC fed with untreated domestic wastewater, where voltage output was 49% higher in light than in dark, suggesting that continuous illumination yields the best performance. However, continuous lighting requires significant energy for artificial illumination, increasing algae cultivation costs. Our hybrid air-cathode/photosynthetic-cathode system mitigates this issue by supplying O_2_ to the cathode even in the dark, reducing diurnal voltage fluctuation and lowering operational costs. Other studies have also noted varied responses to light cycles. Uggetti and Puigagut [59] observed minimal fluctuation (~0.006 V) in a membrane-less pMFC, while Khandelwal et al. [63] reported substantial voltage output during dark periods, even with near-zero DO levels, suggesting nitrate served as an alternative electron acceptor. In our system, nitrate reduction is undesirable, as nitrate is an essential and often limiting nutrient for algal growth.

Light intensity also impacts algal lipid production, a key parameter for biofuel applications. Metsoviti et al. [64] found that higher solar irradiance increased growth rate and lipid content in *C. vulgaris* in an open bioreactor, while Li et al. [65] reported that red–orange light at 45–305 μmol m^−2^ s^−1^ maximized biomass and lipid productivity in *Chlamydomonas reinhardtii*. By operating with half of the cathode exposed to the atmosphere and half submerged in the AC vessel, our MFC-AC design maintains a stable O_2_ supply regardless of light/dark cycle, potentially stabilizing voltage output while supporting algae growth for biofuel production.

### 3.5. Polarization and Maximum Power Density

**Figure 6:**
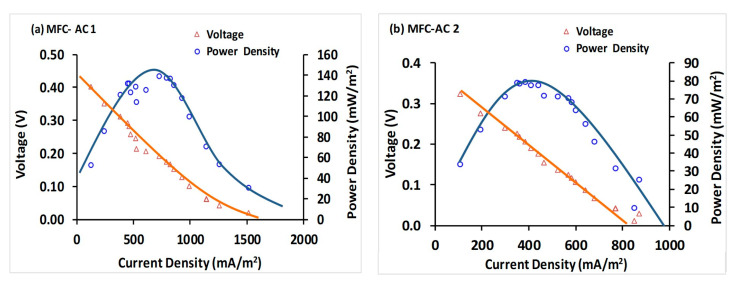
Polarization and power density curves: (**a**) MFC-AC 1; (**b**) MFC-AC 2.

The polarization and power density curves for MFC-AC 1 and MFC-AC 2 are shown in Figure 6a and Figure 6b, respectively. MFC-AC 1 demonstrated substantially higher performance, reaching a maximum power density of 139 mW m^−2^ at a current density of 729 mA m^−2^, with an internal resistance of 390 Ω. In contrast, MFC-AC 2 achieved a maximum power density of 80 mW m^−2^ at a current density of 385 mA m^−2^ and an internal resistance of 802 Ω. The superior performance of MFC-AC 1 can be attributed to the enhanced mass transport properties of its ceramic membrane, which reduced internal resistance and facilitated more efficient electron and ion transfer, ultimately improving energy generation efficiency.

#### 3.5.1. Photosynthetic MFCs (pMFCs)

To place these results in context, Table A1 (Appendix A) compares our findings with other pMFC systems reported in the literature. Del Campo et al. [66] operated a two-chamber pMFC in flow-through mode using fruit-processing wastewater, achieving a maximum power density of 13.5 mW m^−2^. Gajda et al. [67] used nonspecific pond algae in a two-chamber configuration, producing 7.0 mW m^−2^ (based on total electrode area). In a cylindrical two-chamber pMFC with *Chlorella vulgaris*, Gouveia et al. [68] reported 62.7 mW m^−2^, whereas a two-chamber bottle-type MFC with *Scenedesmus obliquus* yielded 30 mW m^−2^ [69]. Using the same algal species, an H-type bottle pMFC reached 153 mW m^−2^ [58]. In a study using an H-type bottle pMFC with a Nafion-117 membrane fed with dairy wastewater, Zieliński et al. [62] reported 91 mW m^−2^ with *Arthrospira platensis* and 34.2 mW m^−2^ with *Chlorella vulgaris*. Kakarla et al. [70] designed a system in which an algal bioreactor was externally connected to an MFC, producing a notably high power density of 630 mW m^−2^ at 2.06 A m^−2^. Ullah et al. [71] employed a two-chamber pMFC with a graphite rod anode inoculated with activated sludge, fed with domestic or sugar-industry wastewater. With *Scenedesmus* sp. in the cathode chamber, the system generated 47.6 mW m^−2^ (domestic wastewater). Similarly, Ullah and Zeshan [61] achieved 81.6 mW m^−2^ using untreated domestic wastewater. A pMFC with *Chlorella vulgaris* produced a power density of 0.85 mW m^−2^ in a study by Colombo et al. [72]. Other recent reports include Wang et al. [60], who obtained 19.76 mW m^−2^ with a cylindrical two-chamber pMFC containing unspecified microalgae, and Wang et al. [73], who reached 42.95 ± 0.12 mW m^−2^ in a two-chamber system with a polyvinyl alcohol proton exchange membrane (PEM), a mixed bacterial culture in the anode, and *C. vulgaris* in BG-11 medium in the cathode. Das et al. [51] developed a ceramic membrane-based pMFC with a carbon felt anode, graphitized bamboo monolith cathode, anaerobic sludge inoculum, and freshwater green microalgae, achieving 85.85 mW m^−2^. Overall, these comparisons highlight the significant influence of system configuration, electrode and membrane materials, and the choice of anolyte and catholyte on pMFC performance. The comparatively strong output of MFC-AC 1, substantially higher than MFC-AC 2 and competitive with other designs in the literature, demonstrates the promise of our integrated MFC-AC approach for improving the overall cost-effectiveness of the system.

#### 3.5.2. MFCs with Ceramic Membrane Separators

Characteristics and performance of ceramic membrane-based MFCs are summarized in Table A2 (Appendix B). Ghadge et al. [74] used two-chamber MFCs with a stainless steel mesh anode and carbon felt cathode. The anode chamber contained synthetic wastewater with sodium acetate as the carbon source and anaerobic sludge from a septic tank as inoculum, while the cathode chamber contained tap water. An MFC with a red soil (rich in Al and Si) separator produced 1.49 W m^−3^ and 51.65 mW m^−2^ (anode area), whereas one with a black soil (rich in Ca, Fe, and Mg) separator produced 1.12 W m^−3^ and 31.20 mW m^−2^ (anode area). Ortiz-Martínez et al. [75] modified terracotta separators with the ionic liquid 1-ethyl-3-methylimidazolium bis(trifluoromethyl sulfonyl)imide ([EMIM][Tf_2_N]) to improve MFC performance. When incorporated into the cathode’s activated layer, this modification significantly enhanced power generation. Pasternak et al. [76] compared various ceramic membranes with a commercially available PEM in single-chamber, air-cathode MFCs fed human urine. Using pyrophyllite, earthenware, mullite, and alumina ceramics, they obtained volumetric power densities of 6.93, 6.85, 4.98, and 2.60 W m^−3^, respectively. You et al. [77] investigated cylindrical air-cathode MFCs with ceramic membrane separators, a carbon fiber veil anode, and a hot-pressed activated carbon cathode. Fed with human urine, white ceramic (brown-spotted) and white ceramic (red-spotted) membranes produced 71.8 and 71.5 W m^−3^, respectively, while red ceramic produced 67.1 W m^−3^. White and red ceramics were more porous with smaller pores, favoring higher performance, while brown ceramics had larger pores, offering greater fouling resistance in long-term operation. Suransh et al. [78] modified a red-soil clayware membrane with montmorillonite and spray-coated Nafion (SMN). The SMN membrane achieved 84.3 mW m^−3^. Das et al. [33] fabricated ceramic membranes by adding 5% goethite to natural clay (5%-G) for two-chamber MFCs with synthetic sucrose wastewater. The 5%-G membrane produced 112.81 ± 8.74 mW m^−2^, slightly higher than Nafion-117 (106.95 ± 5.52 mW m^−2^), with 8.6% higher Coulombic efficiency. The G-5 membrane was estimated to cost five times less than Nafion-117. Salar-García et al. [79] studied urine-fed, single-chamber air-cathode MFCs using ceramic separators with varying iron content and sintering temperatures. The highest output (1.045 mW) was achieved with membranes containing 5.75 vol.% Fe_2_O_3_ and sintered at 1100 °C. Suransh and Mungray [26] tested membranes prepared from red soil, montmorillonite, and vermiculite, obtaining 995.73 ± 49.3 mW m^−3^, outperforming other fabricated membranes in synthetic wastewater-fed single-chamber MFCs. Rao et al. [80] blended potter’s clay with varying proportions of fly ash for two-chamber MFC membranes. The 10% fly ash blend (CFA10) achieved the highest proton mass transfer coefficient (4.32 ± 0.04 × 10^−5^ cm s^−1^) and oxygen mass transfer coefficient (5.13 ± 0.12 × 10^−5^ cm s^−1^), producing 4.57 W m^−3^. Sabina-Delgado et al. [81] prepared ceramic composite membranes from clay and bituminous carbon, achieving 0.699 W m^−3^ at 4.012 A m^−3^, attributed to the high Si and Al content in the activated carbon. de Rosset et al. [28] applied polyvinylidene fluoride (PVDF) nanofiber layers, treated with alkali and rhamnolipids (PVDF-OH/BS), to ceramic membranes to reduce biofouling. The modified membranes achieved 13.8 W m^−3^. Our MFC-AC system produced power outputs comparable to these studies; however, caution is needed when making direct comparisons, as some studies report volumetric power densities while others normalize to cathode area, anode area, or total electrode area.

### 3.6. Algae Growth and Production Rate

**Figure 7:**
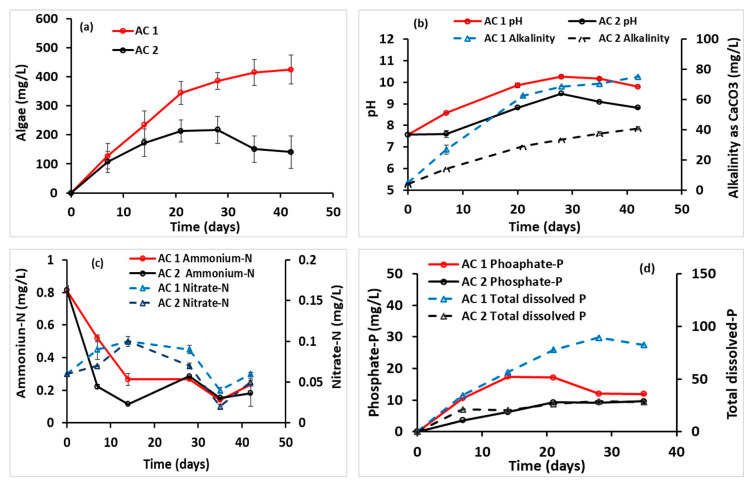
Temporal changes in (**a**) algae concentration, (**b**) pH and alkalinity, (**c**) ammonium-N and nitrate-N concentrations, and (**d**) phosphate-P and total dissolved P in AC 1 and AC 2.

The temporal variations in *C. vulgaris* biomass concentration, pH/alkalinity, and primary nutrients (P and N) in AC 1 and AC 2 are shown in Figure 7. Both cultivators were inoculated on Day 0 with a stock algae suspension containing biomass below the detection limit (<1 mg L^−1^). In AC 1, biomass increased steadily over the first three weeks, after which the growth rate declined, reaching a peak concentration of 425 ± 51 mg L^−1^ by Day 42. In AC 2, growth slowed after the first week, with a lower peak biomass of 218 ± 46 mg L^−1^ before declining. The initial linear growth phase suggests that the algae’s reproductive capacity was not the limiting factor, implying constraints from factors such as light, nutrient availability, or mixing efficiency [82]. The subsequent decline in both cultivators is likely due to nutrient depletion or inaccessibility, or to the accumulation of inhibitory metabolic by-products. Comparable results have been reported in previous pMFC studies. Uggetti and Puigagut [59] observed algal biomass increases from ~120 to 350 mg L^−1^ in a membrane-less pMFC, attributing limited productivity to nutrient deficiencies. Ma et al. [16] achieved substantially higher concentrations (3.5–6.5 g L^−1^) in a tubular two-chamber pMFC with a stainless steel mesh cathode acting as a filter.

To quantify early-phase performance, algae production rates were calculated for the first 14 days. AC 1 exhibited a rate of 234 ± 48 mg L^−1^ d^−1^, which declined after the third week, indicating emerging nutrient limitations. AC 2 recorded a lower rate of 173 ± 48 mg L^−1^ d^−1^, with nutrient deficiency signs appearing as early as the first week. For reference, Powell et al. [82] reported 3.6 mg L^−1^ h^−1^ (86.4 mg L^−1^ d^−1^) in a two-chamber MFC with *C. vulgaris*. Uggetti and Puigagut [59] achieved 29 mg L^−1^ d^−1^ in a membrane-less pMFC, while Hadiyanto et al. [83] reported 55.38 ± 6.39 mg L^−1^ d^−1^ in a two-chamber pMFC with a Nafion-117 membrane. Considerably higher rates have been achieved in other systems. Das et al. [51] reported 560 mg L^−1^ d^−1^ using a bamboo monolith cathode with MnO_2_-doped activated bamboo-leaf biochar (MABB) catalyst. Ullah and Zeshan [61] observed 800 mg L^−1^ d^−1^ in a two-chamber pMFC with untreated domestic wastewater, and Ullah and Zeshan [61] reported a maximum biomass concentration of 5165 mg L^−1^ under similar wastewater-fed conditions. These variations highlight the strong influence of system design, medium composition, and operational conditions on algal productivity. Despite the separation of algae from wastewater, the MFC-AC configuration in this study achieved biomass yields comparable to many previously reported pMFC designs.

A direct comparison of MFC-AC 1 and MFC-AC 2 in terms of maximum power density, algae growth rate, and peak biomass concentration is presented in Table 4. Notably, higher maximum power density was associated with greater algae growth rates and higher biomass concentrations. This relationship can be attributed to improved cathode performance resulting from oxygen production by photosynthetic algae, which serve as the terminal electron acceptor at the cathode. Khandelwal and Lens [24] reported a similar trend in a top–bottom two-chamber pMFC, where algal growth directly enhanced power output via oxygen evolution. Collectively, these findings emphasize the importance of nutrient availability and carefully optimized reactor conditions for achieving both efficient biomass production and enhanced power generation in MFC-AC systems.

### 3.7. pH and Alkalinity

Changes in pH for AC 1 and AC 2 during the experiment are shown in Figure 7b. The initial pH of the algae medium (mean ± standard error) was 7.61 ± 0.08, closely matching the 7.72 value predicted by Visual MINTEQ. During the first few weeks, pH rose substantially, reaching 10.25 ± 0.08 in AC 1 and 9.47 ± 0.02 in AC 2. Around Day 28, pH values in both cultivators stabilized, indicating a plateau phase. This pH increase is consistent with the enhanced uptake of bicarbonate ions (HCO_3_^−^) and oxygen (O_2_) generation via algal photosynthesis. The larger pH shift in AC 1 corresponds with its higher biomass concentration (Figure 7a), suggesting greater photosynthetic activity. Similar pH increases (9.5–10.5) have been reported in membrane-less pMFCs [59]. pH strongly influences algal physiology, including growth, lipid synthesis, and enzymatic activity [84]. While algae generally thrive in acidic to neutral pH, extremely low pH can impair carbon assimilation by reducing carbonate (CO_3_^2−^) and bicarbonate (HCO_3_^−^) availability [84]. In some cases, forced CO_2_ injection has been shown to reduce pH and stimulate lipid synthesis [85].

Alongside nitrogen and phosphorus, carbon (CO_2_) is an essential macronutrient and a potential growth-limiting factor in pMFC systems [84]. In natural waters, alkalinity serves as a measure of available carbonate and bicarbonate species that support algal growth. Alkalinity trends for AC 1 and AC 2 are presented in Figure 7b. Initial alkalinity was ~5 mg L^−1^ on Day 0, but increased steadily over time, reaching 75 ± 0.1 mg L^−1^ in AC 1 and 41 ± 1.2 mg L^−1^ in AC 2 by Day 42. The higher alkalinity in AC 1 parallels its higher pH and greater biomass yield. This rise likely reflects the diffusion of carbonate (HCO_3_^−^, CO_3_^2−^) and phosphate (HPO_4_^2−^) species from the MFC chamber to the AC through the ceramic separator. The greater diffusion rate in AC 1 may have enhanced the supply of carbon and phosphorus, thereby promoting higher algal growth. A comparable trend was reported by Khandelwal and Lens [24], although their system experienced a sharp alkalinity drop from 400 mg L^−1^ to 150 mg L^−1^ within one week, coinciding with slowed algal growth, indicative of carbon limitation. In contrast, the continuous alkalinity increase in our MFC-AC system suggests a sustained influx of carbonate species from the MFC chamber. While algae growth rates still declined after the third week, this was more likely due to depletion of other essential nutrients rather than carbon limitation. Overall, these results highlight the importance of maintaining optimal pH and alkalinity levels to ensure stable carbon availability, sustained photosynthetic activity, and long-term algal productivity in pMFC-based systems.

### 3.8. Ammonium-N and Nitrate-N

Ammonium (NH_4_^+^-N) and nitrate (NO_3_^−^-N) are key nitrogen sources in aquatic systems and are essential for supporting algal growth. Temporal changes in their concentrations for AC 1 and AC 2 are presented in Figure 7c. The initial NH_4_^+^-N concentration in both AC vessels was 0.83 ± 0.02 mg L^−1^. It declined rapidly during the first two weeks and stabilized at approximately 0.2 mg L^−1^ for the remainder of the experiment. This decrease likely reflects nitrification activity, with additional removal through algal assimilation. The data suggest that the rate of NH_4_^+^ diffusion from the MFC chamber into the AC was slower than the combined rates of nitrification and algal uptake. Similar observations were reported by Uggetti and Puigagut [59], who recorded a drop in NH_4_^+^-N from 31 ± 29 mg L^−1^ to 0.5 ± 0.4 mg L^−1^ in an H-type membrane-less pMFC, attributing the decrease to rapid algal consumption and nitrification. In another study, Pei et al. [23] used a tubular two-chamber pMFC with anaerobically digested kitchen waste as the substrate. They found that ~79% of the initial 1550 mg L^−1^ ammonium in the anolyte diffused into the catholyte through a cation exchange membrane (CEM), where most was removed via nitrification, with algal assimilation accounting for ~14.6% of total nitrogen removal.

Initial NO_3_^−^-N concentrations in both ACs were ~0.06 mg L^−1^, rising to ~0.1 mg L^−1^ during the first two weeks. This early increase is consistent with the oxidation of ammonium within the ACs, suggesting that nitrate production (via nitrification and/or diffusion from the MFC) exceeded algal uptake while algal biomass levels were still low. Between Days 14 and 28, algal biomass increased and the system reached a dynamic equilibrium, with nitrate and ammonium supplied through diffusion and nitrification being consumed at comparable rates by the algae. After Day 28, algal nitrate demand exceeded replenishment rates, leading to a steady decline in NO_3_^−^-N to ~0.02 mg L^−1^ by Day 35. This post–Day 28 reduction in nitrogen availability directly aligns with the biomass trends described in Section 3.6, where both AC 1 and AC 2 exhibited slower growth rates after the third week. The close timing of these events suggests that nitrogen limitation was a primary factor in the observed slowdown of algal productivity. Overall, the results indicate that, while initial nitrogen availability supports robust early-stage algal growth, sustained productivity in the MFC-AC system depends on maintaining adequate nitrogen flux from the MFC chamber. These findings highlight the need to consider nitrogen diffusion dynamics when designing and optimizing pMFC systems for integrated algae cultivation and wastewater treatment.

### 3.9. Phosphate-P and Total Dissolved Phosphorus

Temporal variations in phosphate (PO_4_^3−^-P) and total dissolved phosphorus for AC 1 and AC 2 are shown in Figure 7d. The initial total phosphorus concentration in the OECD medium was 0.36 mg L^−1^**,** but the measured value at Day 0 was 0.24 ± 0.07 mg L^−1^, likely due to adsorption onto the internal surfaces of the AC vessels.

Throughout the experiment, PO_4_^3−^-P concentrations were consistently higher in AC 1 than in AC 2. Both systems started at ~0.01 mg L^−1^, but in AC 1, levels rose rapidly to 17.43 ± 1.18 mg L^−1^ by Day 14 before stabilizing, whereas AC 2 increased more gradually, reaching 9.29 ± 0.08 mg L^−1^ by Day 21. The apparent mass flux rate (neglecting algal uptake during the first 14 days) was 0.929 g d^−1^ m^−2^ in MFC-AC 1, compared to 0.329 g d^−1^ m^−2^ in MFC-AC 2. This greater phosphate availability in AC 1 likely supported its higher algal biomass (Figure 7a; see Section 3.6).

After Day 21, PO_4_^3−^-P in AC 1 declined, coinciding with pH reaching 10.25 ± 0.08, above the threshold for calcium phosphate (Ca_3_(PO_4_)_2_) precipitation predicted by Visual MINTEQ. Such precipitation under high pH conditions (9.0–10.5) has also been observed by Uggetti and Puigagut [59], who reported a decrease in PO_4_^3−^-P from 4.4 ± 0.6 mg L^−1^ to 0.0 ± 0.2 mg L^−1^ in a membrane-less pMFC, attributing it to precipitation and biomass uptake.

By Day 28, total dissolved phosphorus reached 89 mg L^−1^ in AC 1 and 29 mg L^−1^ in AC 2. Since there were no other phosphorus inputs, this rise indicates that diffusion from the MFC wastewater was the primary source. Notably, total dissolved phosphorus consistently exceeded PO_4_^3−^-P levels, suggesting the presence of other phosphorus forms, likely organic phosphorus compounds released from algal cells or diffused from the MFC chamber. As with orthophosphate, the higher total phosphorus concentration in AC 1 likely contributed to its greater biomass level.

The slowing of phosphate accumulation in AC 1 after the third week coincided with the pH plateau at ~10, supporting the interpretation that precipitation reduced dissolved phosphorus availability. Taken together with the nitrogen dynamics described in Section 3.8, these results indicate that the post–third-week slowdown in algal growth (Section 3.6) was likely driven by the combined effects of nitrogen depletion and phosphorus precipitation. Maintaining an optimal pH and ensuring sustained availability of both nitrogen and phosphorus are therefore critical for long-term algal productivity and overall efficiency in MFC-AC systems.

### 3.10. Inorganic Species

**Figure 8:**
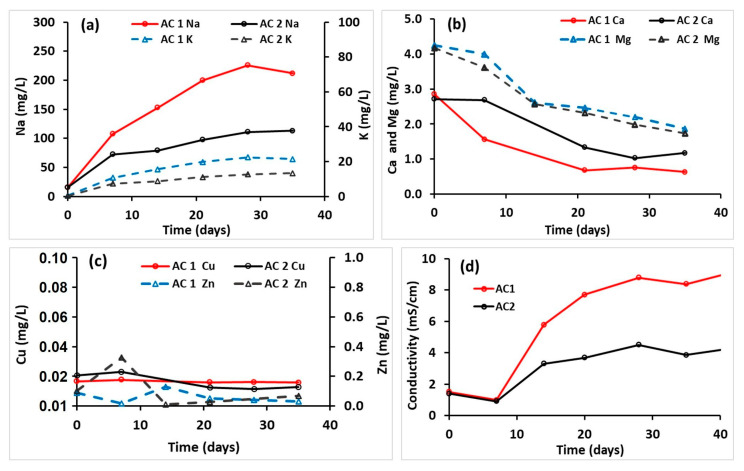
Temporal changes in the concentration of (**a**) sodium and potassium, (**b**) calcium and magnesium, (**c**) copper and zinc, and (**d**) conductivity in AC 1 and AC 2.

Figure 8 shows the temporal concentration profiles of sodium (Na^+^), potassium (K^+^), calcium (Ca^2+^), magnesium (Mg^2+^), copper (Cu^2+^), zinc (Zn^2+^), and conductivity in AC 1 and AC 2. Sodium, known for its high mobility and chemical inertness in aqueous systems, was initially 15.9 ± 0.2 mg L^−1^ in both reactors. In AC 1, Na^+^ concentrations rose sharply, peaking at ~225 mg L^−1^ by Day 28, while AC 2 peaked at ~110 mg L^−1^. Potassium levels, though roughly an order of magnitude lower, showed similar trends. Because K^+^ is an essential macronutrient for algal metabolism, its higher levels in AC 1 may have contributed to the greater biomass yield reported in Section 3.6.

Initial Ca^2+^ concentrations (~2.8 mg L^−1^) decreased to 0.6 mg L^−1^ in AC 1 and 1.2 mg L^−1^ in AC 2 by Day 35. This decline is consistent with early adsorption to vessel walls and subsequent precipitation as calcium carbonate (CaCO_3_) and calcium phosphate (Ca_3_(PO_4_)_2_) once the pH rose. The higher pH in AC 1 appears to have resulted in a lower calcium level in AC 1 than in AC 2. Mg^2+^ concentrations fell from ~4 mg L^−1^ to 2 mg L^−1^ over the same period, also likely due to precipitation at elevated pH. Visual MINTEQ simulations indicate that CaMg(CO_3_)_2_ formation begins at pH 9.5–10. Neither ion showed evidence of significant net diffusion from the MFC into the AC water.

Copper present in the OECD medium as a trace micronutrient began at 0.020 ± 0.002 mg L^−1^ and declined slightly to 0.015 ± 0.001 mg L^−1^ by Day 35, with no significant differences between ACs. The reduction may be due to precipitation (e.g., CuFe_2_O_4_(s)) or algal uptake. Zn concentrations were roughly an order of magnitude higher than Cu, but no clear time trend emerged, and no AC-specific differences were evident.

Initial conductivity was 1.43 ± 0.03 mS cm^−1^ in both ACs. Values rose rapidly after the first week, stabilizing by Day 28 at 8.78 ± 0.19 mS cm^−1^ in AC 1 and 4.50 ± 0.01 mS cm^−1^ in AC 2, paralleling the Na ion profiles. The higher conductivity in AC 1 indicates greater ion flux through its ceramic separator, which likely enhanced nutrient supply and early-stage growth (Section 3.6).

These inorganic ion dynamics reinforce the nutrient-limitation sequence observed in this study. In the early phase, greater Na^+^, K^+^, and total ion flux (indicated by conductivity) in AC 1 likely supported its faster biomass accumulation. However, by the third week, precipitation of phosphate, combined with nitrogen depletion (Section 3.8), constrained further growth of algae. This supports the conclusion that the post–third-week slowdown in algal biomass production was driven by a convergence of nutrient limitations rather than a single factor. Optimizing ceramic separator ion permeability while controlling pH could help maintain balanced nutrient availability and extend the high-growth phase in MFC-AC systems.

### 3.11. Overall Outlook

Between the two configurations tested, MFC-AC 1 consistently outperformed MFC-AC 2 in voltage output, power density, and algal biomass productivity. This advantage was primarily due to the higher ion permeability of MFC-AC 1’s ceramic membrane, which enhanced nutrient transfer, supported faster algal growth, and lowered internal resistance. These results underscore the critical role of membrane properties and reactor design in the integrated MFC-AC system.

Beyond performance, the present work also demonstrated the viability of using low-cost, natural materials, such as clay for ceramic membranes and bamboo charcoal for anodes, to address the cost constraints that limit large-scale MFC deployment [51]. Leveraging inexpensive, renewable materials reduces the economic barrier to scaling, while maintaining functional efficiency in both electricity generation and algal cultivation.

The integrated MFC-AC approach has the potential to contribute to three sustainability targets:

- Renewable energy generation through microbial fuel cells.
- Sustainable wastewater treatment via nutrient recovery.
- High-quality algal biomass production suitable for biofuel or other value-added applications.

Advances in membrane engineering, ion transport optimization, and reactor integration will be essential to move from lab-scale proof-of-concept to practical, deployable systems.

## 4. Conclusions

Algae-based biofuel production must achieve both economic and environmental sustainability to be viable, requiring improvements from cultivation to refining [13]. This study’s primary goal was to develop a cost-effective, sustainable algae cultivation technology by integrating an algae cultivator (AC) with a microbial fuel cell (MFC), using wastewater as a renewable nutrient and energy source.

The MFC-AC system incorporated a ceramic membrane interface, hybrid cathode design, and nutrient recovery from wastewater, achieving cost savings through:

- Separation of wastewater from clean algal culture, reducing pretreatment needs.
- A photosynthetic–air-cathode configuration that minimized diurnal voltage fluctuations and enabled electricity generation even during dark periods, thereby eliminating reliance on artificial lighting.
- Use of low-cost, renewable natural materials (clay membrane, bamboo charcoal electrodes).

Key findings comparing MFC-AC 1 (high-permeability membrane) vs. MFC-AC 2 (lower-permeability membrane) are:

- Na^+^ mass flux rate: 0.424 vs. 0.315 g d^−1^ m^−2^;
- Average voltage: 0.346 V vs. 0.270 V;
- Maximum power density: 139 ± 0.16 mW m^−2^ vs. 80 ± 0.01 mW m^−2^;
- Internal resistance: 390 Ω vs. 802 Ω;
- Peak algal biomass: 425 ± 51 mg L^−1^ vs. 218 ± 46 mg L^−1^;
- Algal growth rate: 234 ± 48 mg L^−1^ d^−1^ vs. 173 ± 48 mg L^−1^ d^−1^;
- Apparent PO_4_^3−^-P flux: 0.929 vs. 0.329 g d^−1^ m^−2^.

The superior performance of MFC-AC 1 highlights membrane ion permeability as a decisive factor in enhancing nutrient transport, sustaining biomass growth, and maximizing electrochemical output. The system successfully integrates wastewater treatment, producing clean algal biomass while generating renewable electricity.

## 5. Future Work

While this study established the feasibility of integrated MFC-AC operation, several open questions and opportunities remain:

- Causality between power generation and biomass growth: Although positive correlations were observed, it is unclear whether higher electrochemical output stimulates algal productivity directly or is simply a co-benefit of nutrient availability (e.g., via enhanced ion transport). Controlled studies varying membrane conductivity independent of nutrient concentrations could address this.
- Advanced membrane engineering: Development of ceramic membranes with tunable ion permeability and optimized pore structure could allow nutrient flux control. Electrical conductivity measurements could serve as a rapid, low-cost diagnostic for membrane performance.
- Long-term operational stability: Multicycle experiments are needed to assess fouling, mechanical durability, and performance drift in both the membrane and electrodes.
- Nutrient-limitation management: Strategies to prevent late-stage nitrogen limitation, such as staged nitrogen (e.g., ammonia, nitrate) dosing, should be explored to maintain steady biomass output.

Ultimately, a deeper understanding of the interplay between ceramic ion transport, water chemistry (e.g., pH dynamics), and algal photosynthetic activity will be central to scaling MFC-AC systems for sustainable energy, nutrient recovery, and wastewater treatment at practical scales.

## Figures and Tables

**Table 1:** Chemical characteristics of the feed potato-process wastewater.

Parameters	Unit	Values	STER
pH		6.84	0.02
Alkalinity as CaCO_3_	mg L^−1^	622	--
Ammonium-N	mg L^−1^	0.07	0.00
Nitrate-N	mg L^−1^	0.06	0.00
Phosphate-P	mg L^−1^	1739	115
Chemical Oxygen Demand (COD)	mg L^−1^	2875	44
Conductivity	mS cm^−1^	62.73	0.38
ICP-MS Analysis			RSTD
Na	mg L^−1^	1056	0.00
K	mg L^−1^	378	0.72
P	mg L^−1^	3028	1.26
Ca	mg L^−1^	4.23	1.53
Mg	mg L^−1^	22.98	0.17
Mn	mg L^−1^	0.086	1.84
Al	mg L^−1^	0.394	2.39
Ba	mg L^−1^	0.026	0.99
Cr	mg L^−1^	0.011	2.74
Cu	mg L^−1^	0.069	1.81
Fe	mg L^−1^	0.287	4.52
Sr	mg L^−1^	0.073	1.49
Zn	mg L^−1^	0.130	1.49

STER, standard error; RSTD, relative standard deviation; --, no data.

**Table 2:** SEM/EDS averaged results of multiple area measurements on natural Peruvian clay used for manufacturing ceramic membranes. Data are recorded as non-normalized stoichiometric oxide %.

Major Elements	Stoichiometric Oxide %
Na_2_O	1.48
MgO	5.47
Al_2_O_3_	13.73
SiO_2_	43.88
K_2_O	2.66
CaO	10.21
TiO_2_	0.62
FeO	4.89

Analysis conditions: Accelerating voltage: 20 kV; working distance: 10 mm; horizontal field width (HFW) of EDS collection area: ~300 µm; X-ray data collection: 500 s.

**Table 3:** LA-ICP-MS averaged results of replicate (4×) laser ablations on ceramic separators.

Analyte	Concentration (µg/g)	Analyte	Concentration (µg/g)
^7^Li	32.58	^115^In	0.06
^9^Be	1.41	^118^Sn	1.37
^11^B	43.93	^121^Sb	2.43
^31^P	427.12	^133^Cs	0.07
^45^Sc	8.06	^137^Ba	403.66
^51^V	77.44	^139^La	19.34
^52^Cr	33.54	^140^Ce	33.97
^55^Mn	540.89	^141^Pr	3.91
^59^Co	8.53	^146^Nd	13.94
^60^Ni	20.2	^147^Sm	2.77
^65^Cu	18.7	^153^Eu	0.79
^66^Zn	80.33	^157^Gd	2.67
^69^Ga	11.83	^159^Tb	0.41
^72^Ge	2.47	^163^Dy	2.34
^75^As	31.91	^165^Ho	0.48
^82^Se	0.58	^166^Er	1.31
^85^Rb	38.73	^169^Tm	0.2
^88^Sr	222.89	^172^Yb	1.32
^89^Y	12.67	^175^Lu	0.2
^90^Zr	18.09	^208^Pb	2.84
^93^Nb	8.14	^209^Bi	0.3
^95^Mo	1.47	^232^Th	6.07
^107^Ag	0.44	^238^U	2.26
^111^Cd	0.52		

Analysis conditions: 193 nm (excimer) laser ablation device, operating at 65% laser power, Fluence: 4.3 J/cm^2^, spot diameter: 30 µm, raster speed: 25 µm/s^−1^. Mass spectrometer: 0.1 s dwell time per mass, 55 elements.

**Table 4:** Comparison of MFC-AC 1 and MFC-AC 2 in terms of initial algae growth rate, peak algae concentration, maximum power density, and maximum flux rate.

System	Algae Growth Rate ^a^	Algae Conc. at Peak ^b^	Max. Power Density ^c^	Ceramics Separator Mass Flux Rate ^d^
Unit	(mg L^−1^ d^−1^)	(mg L^−1^)	(mW m^−2^)	(g d^−1^ m^−2^)
MFC-AC 1	234 ± 48	424.8 ± 51	138.78 ± 0.16	0.424
MFC-AC 2	173 ± 48	217.5 ± 46	79.51 ± 0.0	0.315

^a^ Determined for the first 14 days. ^b^ Determined at the peak. ^c^ Normalized to the surface area of the cathode. ^d^ Na^+^ was used as a model solute.

## Data Availability

The original contributions presented in this study are included in the article. Further inquiries can be directed to the corresponding author.

## Abbreviations

The following abbreviations are used in this manuscript:

BBM	Bold basal medium
CE	Coulombic efficiency
CEM	Cation exchange membrane
PEM	Proton exchange medium
pMFC	Photosynthetic microbial fuel cell

## Appendix A

**Table A1:** A summary of certain studies evaluating the performance of photosynthetic microbial fuel cells (pMFCs).

MFC Type	Anode Chamber, Anolyte, Electrode, EAB	Cathode Chamber, Catholyte, Electrode	Separator	Algae Species/Inoculum	Performance	Comment	Reference
Two-chamber Honeycomb MFC	Anode chamber (5 × 5 × 8 cm); Anolyte: domestic wastewater; Inoculum: mixed bacterial culture.	Cathode chamber (5 × 5 × 6 cm); Catholyte: BG 11 medium.	Polyvinyl alcohol membrane.	*Chlorella vulgaris*	Max. PD: 42.95 ± 0.12 mW m^−2^; Peak CD: 329.0 mA^−2^.	Anolyte was continuously recirculated.	[73]
H-type two-chamber pMFC	A 1000 mL glass bottle (each chamber); Catholyte: synthetic medium + diluted effluent from the anode chamber; Anolyte: dairy wastewater; Inoculum: anaerobic sludge;	Anode: carbon cloth; Cathode: carbon rod;	PEM (Nafion 117)	*Chlorella vulgaris*; *Arthrospira pltensis*	91 mW m^−2^ with *Arthrospira pltensis*.	Operation mode: batch; significant diurnal fluctuation in voltage;	[62]
Top–Bottom Two-chamber MFC	Graphite rod anode; Inoculum: activated sludge from a dairy WWTP; Annolyte: synthetic medium.	Graphite rod cathode; Catholyte: modified BG-11 media.	Clay membrane separator.	Unspecified microalgae culture.	Max. PD: 24.99 mW m^−3^; Electrical energy recovery: 0.1 kWh m^−3^; Total energy recovery: 7.25 kWh m^−3^.	Light source: LED emitting 450 lumens/m^−2^.	[24]
Cylindrical two-chamber MFC	A cylindrical chamber (0.25 L); Graphite rod anode; Inoculum: mixed culture from anaerobic digester; Anolyte: synthetic biogas slurry.	A cylindrical chamber (0.25 L); Graphite rod anode; Catholyte: effluent from the anode chamber.	Unspecified PEM.	Unspecified microalgae culture.	Max. PD: 19.76 mW m^−2^; average output voltage of 0.412 V.	Flow-through operation (HRT: 24 h); Light intensity: 5500 lx.	[60]
Two-chamber MFC	Anode chamber (WV: 1 L); Anolyte: untreated domestic wastewater; Inoculum: activated sludge.	Cathode chamber (WV: 1 L); Catholyte: untreated domestic wastewater.	CEM (CMI-7000)	*Scenedesmus* sp.	Max. PD: 81.6 mW m^−2^; Avg. Max. voltage: 414 mV; Average biomass productivity: 800 mg L^−1^ d^−1;^ Max. algae conc. 5165 mg L^−1^.	Operation mode: batch.	[61]
Two-chamber MFC	Anode chamber (WV: 900 mL) with a photobioanode.	Cathode chamber (WV: 900 mL); Carbon felt cathode.	PEM (Nafion 117)		voltage produced: 2.5 V; produced current: 2.8 A.	Operation: fed-batch mode.	[86]
Two-chamber MFC	A chamber (WV: 1 L); graphite rod anode; Inoculum: decanted activated sludge; Anolyte: domestic wastewater or sugar industry wastewater.	A chamber (WV: 1 L); graphite rod cathode.	CEM (CMI-7000S)	*Scenedesmus* sp.	Max. PD: 47.6 mW m^−2^.	Operation: batch mode; p-MFC yielded lower internal resistance than MAMFC.	[71]
Two-chamber MFC	A polyacrylic chamber (3 L working volume); Graphite rod anode.	A polyacrylic chamber (3 L working volume); Graphite rod cathode.	PEM (Nafion 117)	*Spirulina platensis*	Max. PD: 14.47 ± 0.7 mWm^−2^.	Algae productivity: 55.38 ± 6.39 mg L^−1^ d^−1^.	[83]
Rectangular two-chamber p-MFC with a gas exchange tube	Rectangular chamber; 100 mL working volume; Graphite felt anode; Anolyte: synthetic solution.	A rectangular chamber (100 mL working volume); Graphite felt cathode.	PEM (Nafion 117)	*Chlamydomonas reinhardtil* cc-503	Max. PD: 15.21 W m^−3^; Max. current density: 39 A m^−3^.	Operation mode: fed-batch; µ = 0.284 d^−1^; COD removal: 73.30%; CE: 9.068%.	[20]
Tubular-type MFC with multiple electrodes in the cathode chamber	A cube chamber (working volume of 280 mL); Carbon fiber brush anode; Inoculum: Anolyte: domestic wastewater.	A glass bottle (WV: 620 mL); Cathode: carbon fiber brush; Inoculum: mixed culture isolated from municipal wastewater; Catholyte: BBM; Illumination: 7000 lx.	PEM (CMI-7000)	Algae culture isolated from wastewater.	Produced voltage: 315 mV.	Operation mode: Continuous flow.	[87]
Two-chamber p-MFC	A chamber (140 mL); Graphite fiber brush anode; Algae biomass + synthetic anolyte; inoculated with anaerobic soil sediments.	A chamber (140 mL); Graphite felt cathode; Catholyte: BG-11 media supplemented with trace elements.	PEM (Nafion 117)	*Microcystis aeruginosa*	Max. PD: 83 mW m^−2^; Max current density: 672 mA m^−2^; CE of 7.6%.	COD removal: 67.5 ± 1%	[88]
Two-chamber MFC	A cube chamber (WV: 205 mL); Carbon fiber brush anode; Inoculum: domestic wastewater; Anolyte: GM media.	A cube chamber (WV: 205 mL); Carbon fiber brush cathode; Photon flux: 68 µE m^−2^ s^−1^.	PEM (Nafion 117)	Mixed microalgae culture (*Chlorella* sp., *Desmodesmus* sp., *Scenedismus* sp.) isolated from municipal wastewater.	Sustainable voltage: 0.31 V.	Max. ammonium-N removal rate: 22.7 mg L^−1^ d^−1^; Ammonium-N removal: 95.5%.	[89]
Airlift type MFC (AL-PMFC)	A 380 mL chamber; carbon brush anode; Anolyte: synthetic medium; inoculated anaerobic digester sludge; operated in a batch mode.	An air-lift type chamber (380 mL) with a baffle; Carbon fiber cloth (0.1 mg cm^−2^ pt coated) cathode; Catholyte: modified B-11 medium; Operation: batch mode.	PEM (Nafion 117)	*Chlorella vulgaris*	Max PD: 5.94 W m^−3^; Max. net energy: 2.701 kWh m^−3^; Maximum CO_2_ fixation rate: 1292.8 mg L^−1^ d^−1^; Max lipid productivity: 234.3 mg L^−1^ d^−1^.	CO_2_ fixation rate: algae productivity: 835.7 mg L^−1^ d^−1^	[90]
Two-chamber MFC	Anolyte: Swine wastewater; pH: 7.0–7.5; TOC: 780–800 mg/L; ammonium-N: 320–330 mg/L; Total-N: 330–350 mg/L. The wastewater was filtered.	Catholyte: swine wastewater (3-fold diluted) with microalgae.	CEM	*Chlorella vulgaris*	Max. PD: 3720 mW/m^3^; Percent removal of ammonia-N (85.6%), total-N (70.2%), TOC (93.9%).	*C. vulgaris* had direct contact with swine wastewater.	[91]
Tubular two-chamber p-MFC	A tubular chamber (350 mL working volume); Anode: carbon cloth interwoven with titanium wires; Anolyte: anaerobically digested kitchen waste.	A chamber (1500 mL working volume); Cathode: a layer of carbon cloth was wrapped around the CEM.	CEM	*Golenkinia* sp. SDEC-16	Energy output: 0.57 kWh m^−3^; Algae mass conc: 0.94 g L^−1^.	CEM was wrapped around the tubular anodic chamber; Ammonium transport rate: 2.3–2.7 g m^2^ d^−1^	[23]
Two-chamber p-MFC	A 1 L glass bottle; Graphite plate anode; Anaerobic bacterial consortium from domestic and industrial dumps; Blue dye brl	A glass bottle (1 L); Graphite plate cathode; Catholyte: effluent from chocolate industry	PEM (Nafion 117)	*Chlorella vulgaris*	Max. PD: 327.67 mW m^−2^; Max. CD: 343.47 mA m^−2^.	Illuminance: 75,000 lx	[92]
Cylindrical two-chamber p-MFC (algae biofilm)	Anode electrode: carbon cloth interwoven with titanium wire; Anolyte: Domestic wastewater; in a batch mode.	Cathode electrode: carbon cloth woven with titanium; Catholyte: Domestic wastewater; Operation: a batch mode.	Compressed glass wool thickness 20, diameter 110 mm).	*Scenedesmus quadricauda* SEDC-8	PD: 62.93 mW m^−2^.	Lipid productivity: 6.26 mg L^−1^ d^−1^.	[93]
Two-chamber LEA-fed MFC	A 100 mL chamber; Graphite felt anode; EAB: microbial consortia from cow manure; Substrate: lipid-extracted algae (LEA); run in a fed-batch mode.	A chamber (100 mL); Graphite felt cathode; Catholyte: BG 11 media; Operation: a fed-batch mode.	PEM (Nafion 117)	*Chlorella vulgaris*	PD: 2.7 W m^−3^; Algae productivity: 0.028 kg m^−3^ d^−1^.	Electricity generation: 0.0136 kWh kg^−1^ COD d^−1^; Algae oil energy: 0.0782 kWh m^−3^ d^−1^.	[63]
Air-exposed single-chamber MFC	A 125 mL chamber; in a fed-batch operation; Carbon fibre anode; Anolyte: dye textile wastewater.	Microalgae attached on the cathode; Carbon fibre cathode.	Cellophane	*Chlorella vulgaris*	Max. PD: 123.2 ± 27.5 mW m^−3^.	12 h/12 h: light/dark cycle.	[94]
Tubular two-chamber MFC	A tubular anodic chamber (79 mL effective volume); Carbon brush anode.	A tubular cathodic chamber inside the anodic chamber; Stainless steel mesh cathode.	PEM (Nafion 117)	Dominantly *Chlorella* sp. community	Max. CD: ~200 mA m^−2^.	Stainless steel mesh cathode also served as filter; biomass concentration: 3.5–6.5 g L^−1^.	[16]
H-type cylinder membrane-less MFC	A plexiglass cylinder (400 mL); Anode: Graphite rod; Inoculum: digester sludge; Anolyte: primary sludge.	A plexiglass cylinder (400 mL); Cathode: graphite rod; Catholyte: filtered primary settled wastewater.	Glass-wool	Mixed algae consortia culture	Produced voltage: ~15 mV; Algae production rate: 29 mg/L·d.	Voltage change between light and dark was ~6 mV.	[59]
A tubular MFC followed by a membrane photoreactor	A CEM tube (1.75 L working volume); Carbon brush anode; Anolyte: Synthetic solution; Inoculum: anaerobic sludge from municipal WWTP.	Cathode: carbon cloth coated with activated carbon; Inoculum: green algae collected from a local water pond.	CEM (Ultrex CMI-7000)	Mixed algae consortia culture	Max. PD: 2.5 W m^−3^; The highest energy production: 0.205 kWh m^−3^.	Synthetic solution first flowed through the MFC anode and then was fed into the photobioreactor.	[95]
Two-chamber MFC with a biomass harvesting photobioreactor.	An anode chamber (25 mL); Anode: carbon fibre veil; Inoculum and Anolyte: activated anaerobic sludge.	A cathode chamber (25 mL); Cathode: carbon fibre veil; Inoculum: pond water.	CEM (VWR International).	Mixed culture isolated from pond water.	128 µW by algae-based cathode.	Operation mode: a closed-loop recirculation.	[96]
Two-chamber p-MFC	A cubical chamber (600 mL); Carbon brush anode; Anolyte: Synthetic municipal wastewater.	Air-lift-type cubical chamber (500 mL); Carbon fiber cloth cathode; Catholyte: anode effluent.	PEM (Nafion 117)	*Chlorella vulgaris* (ESP-6)	Max. PD: 972.5 mW m^−3^;	Removal of COD: 86.69%; Ammonium-N: 69.24%; phosphorus (69.24%).	[22]
Two-chamber p-MFC	A cube chamber (working volume of 205 mL); Carbon fiber brush anode; Inoculum: wastewater + acetate (2 g L^−1^); Anolyte: GM media + acetate (2 g L^−1^).	A glass bottle (580 mL working volume with 30 mL head space); Cathode: carbon cloth coated with Pt (0.5 mg cm^−2^); Catholyte: BBM.	PEM: Nafion-117	Mixed culture isolated from municipal wastewater.	Max. PD: 0.63 W m^−2^ at 2.06 A m^−2^.	An algae bioreactor (ABR) was externally connected to air-cathode MFC; Illumination: 5000 lumens.	[70]
Two-chamber bottle-type MFC	A glass bottle (250 mL working volume); Plain carbon paper anode; Anolyte: started from municipal wastewater + Acetate, changed to GM media.	A glass bottle (250 mL working volume); Carbon brush cathode; Catholyte: BBM.	Unspecified membrane	*Scenedesmus obliquus*	Max. PD: 30 mW m^−2^.	MFC was operated at 30 ± 1 °C; Light of 5000 lumens.	[69]
H-type bottle MFC	A bottle-type chamber (estimated volume: 125 mL); Plain carbon paper anode; Anolyte: domestic wastewater, later changed to GM media.	A bottle-type chamber (~125 mL); Pt (0.5 mg/cm^2^) coated carbon paper cathode; Catholyte: BBM and sodium carbonate.	PEM (Nafion 117)	*Scenedismus obliquus*	Max. PD: 153 mW m^−2^; voltage of 0.47 ± 0.03 V.	MFC was operated at 30 ± 1 °C; Light of 5000 lumens.	[58]
Cylindrical two-chamber MFC	Cylindrical chamber (50 mL); plain graphite anode; Bacterial consortium.	Cylindrical chamber (50 mL); plain graphite cathode (0.5 mg/cm^2^ Pt coating).	PEM (Nafion 117)	*Chlorella vulgaris*	Max. PD: 62.7 mW m^−2^.	Light intensity range: 26–96 µE m^−2^ s^−1^.	[68]
H-type two-chamber MFC	A chamber (270 mL); Toray carbon paper anode; Anolyte: synthetic medium; Inoculum: wastewater; operation: a batch mode.	A chamber (270 mL); Toray carbon paper cathode; Catholyte: ferricyanide in phosphate buffer; operation: a batch mode.	PEM (Nafion 117)	*Scenedismus obliquus*	Max. PD: 102 mW m^–2^ (951 mW m^–3^) at current density of 276 mA m^−2^.	COD reduction: 74% for 150 h operation.	[97]
Two-chamber MFC	A chamber (25 mL); carbon fibre veil (20 g/m^2^ carbon loading) anode; Inoculum: anaerobic activated sludge.	A chamber (25 mL); carbon fibre veil (20 g/m^2^ carbon loading) cathode; Inoculum: pond water.	Unspecified CEM	Nonspecific pond algae (mixed consortia).	Max. PD: 7.00 mW m^−2^.	14 h light/10 h dark cycle; catholyte was circulated; anode chamber was operated in a fed-batch mode.	[67]
Two-chamber-MFC	A chamber (800 mL); Cathode: Toray carbon cloth with 10% Teflon; Inoculum: activated sludge from municipal WWTP; Anolyte: synthetic fruit processing industry effluent.	An 800 mL chamber; Anode: Toray carbon cloth with 10% Teflon); Catholyte: BBM	PEM (Sterion)	*Chlorella vulgaris*	Max. PD: 13.5 mW m^−2^.	Cathode chamber was illuminated for 12 h/day; Bubbling CO_2_ for 30 min d^−1^.	[66]
Sequential anode–cathode configuration MFC with a photobioreactor	A stainless steel cylinder; Carbon brush anode; Anolyte: primary clarifier effluent.	A plastic cylinder; Carbon brush cathode; Catholyte: effluent from the anode chamber.	CEM	Mixed culture from domestic wastewater.	Max. power density: 20.3 W m^−3^.	Continuous feed operation.	[21]
Two-chamber MFC	A half-circular column (400 mL) with a vent at the top; Carbon felt anode.	A half-circular column (400 mL) with a vent at the top; Carbon fiber cloth (0.1 mg cm^−2^ Pt coating).	CEM (Ultrex CMI-7000)	*Chlorella vulgaris* (Immobilized)	Max. power density: 2485.35 mW m^−3^; CE: 9.40%; COD removal: 84.8%.	Two chambers were connected through a tube for CO_2_ supply from anode chamber to cathode chamber.	[98]
Two-chamber MFC	A cubic chamber (WV: 75 mL); Graphite plate anode; Anolyte: modified Zehnder medium.	A cubic chamber (WV: 75 mL); Graphite plate cathode; Catholyte: K-ferricyanide in phosphate buffer.	CEM (Ultrex CMI-7000)	*Chlorella vulgaris*; *Dunalieella tertiolecta*	Max. PD: 15.0 mW m^−2^ (*C. vulgaris*); 5.3 mW m^−2^ (*D. tertiolecta)*).	Butanol was produced in the anodes of the MFCs.	[99]
Single-chamber tubular MFC	A PVC tube (EV: 170 mL); Graphite felt anode; Inoculum: anaerobic sludge from local WWTP; Operation: a fed-batch mode; Anolyte: domestic wastewater.	A PVC tube (EV: 170 mL); A cloth cathode with 5.0 ± 0.1 mg cm^−2^ MnO_2_ loading.	None	Blue-green algae	Max. PD: 114 mW m^−2^ at 0.55 mA m^−2^; CE of 28.2%.	Removal efficiencies: total COD (78.9%); soluble COD (80.0%); total N (91.0%); ammonium N (96.8%) in 12 days.	[100]
Sediment-type single-chamber MFC	A glass bottle (500 mL); Carbon paper anode; Lake sediments; Anolyte: synthetic wastewater.	No cathode chamber; A piece of 5% wet-proofed carbon paper (0.5 mg Pt cm^−2^); Anolyte: synthetic wastewater.	None	*Chlorella vulgaris*	Max. PD: 68 ± 5 mW m^−2^.	Biomass: 0.56 ± 0.02 g L^−1^.	[101]
Two-chamber MFC	A half-circular column; carbon fiber brush anode; Inoculum: mixed culture originally from domestic wastewater; Substrate: glucose.	A half-circular column; Pt-coated carbon cloth cathode; Anolyte: NBS amended with BG11.	CEM (Ultrex CMI-7000)	*Chlorella vulgaris*	Max. PD: 5.6 W m^−3^.	Carbon capture efficiency: 94 ± 1%.	[102]
Two-chamber MFC	A bottle (WV: 100 mL); Anolyte: modified BBM with 0.02 M potassium ferrocyanide.	A bottle (WV: 100 mL); Catholyte: modified BBM.	A salt bridge filled with saturated KCl, jellified with agar.	*Chlorella vulgaris*	1.0 µA mg^−1^ of cell dry weight; 2.7 mW m^−2^ cathode area; Max. cell growth rate: 3.6 mg of cells L^−1^ h^−1^.	Operation mode: batch; Methylene blue mediator.	[82]

Abbreviations: ABR—algae bioreactor; BBM—bold basal medium; CD—current density; CE—coulombic efficiency; CEM—cation exchange membrane; EAB—electroactive bacteria; EV—empty volume; GM—growth medium; HRT—hydraulic retention time; LEA—lipid-extracted algae; MAMFC—mechanically aerated MFC; PEM—proton exchange membrane; PD—power density; WV—working volume; WWTP—wastewater treatment plant.

## Appendix B

**Table A2:** A summary of certain studies evaluating the performance of ceramic membrane-based microbial fuel cells.

MFC Type	Characteristics of Ceramics	Type of Fuel (Media, Substrate, Anolyte)	Experimental (Operating) Conditions	MFC Performance	Reference
Single-chamber MFC, air-cathode configuration; Anode: carbon veil; Cathode: activated carbon coated with PTFE.	White clay; Ceramic membrane integrated with PVDF treated with alkaline and rhamnolipids (PVDF-OH/BS).	Anolyte: mineral salt medium; Inoculum: activated sludge.	Fed-batch mode; 25 °C.	Ceramic integrated with PVDF-OH/BS: 13.8 W m^−3^, 30% higher than that of unmodified ceramic membrane.	[28]
Cylinder-based MFCs, assembled as cascade; Anode: carbon veil; Cathode: carbon veil coated with AC/PTFE.	Earthenware ceramic cylinders.	Anolyte: urine.	Continuous flow mode; HRT: 12 h/each module; Flow rate: 0.66 mL min^−1^; 22 ± 1 °C.	Energy output: 0.75 ± 0.04 kJ d^−1^.	[103]
Two-chamber MFC.	Natural red clay with bone char, coconut shell activated carbon, or bituminous activated carbon.	Anolyte: domestic wastewater with sodium acetate; Inoculum: domestic wastewater.	Batch mode (3 cycles); Room temperature.	Clay with bituminous carbon: 0.699 W m^−3^ at 4.012 A m^−3^.	[81]
Two-chamber MFC; Anode: carbon felt; Cathode: carbon felt.	Potter’s clay blended with fly ash with varying proportions.	Anolyte: synthetic wastewater with sodium acetate; Inoculum: anaerobic sludge; Catholyte: tap water with continuous aeration.	Batch mode.	The MFC with the ceramic separator made from clay blend with 10% flyash (CFA_10_) produced a peak power output of 4.57 W m^−3^.	[80]
Cylinder-based MFCs, assembled as cascade; Anode: carbon veil; Cathode: carbon veil coated with AC/PTFE	Earthenware ceramic cylinders;	Anolyte: artificial urine.	Continuous flow mode.	32.2 ± 3.9 W m^−3^.	[104]
Single-chamber air-cathode MFC; Anode: carbon felt; Cathode: carbon felt.	Red soil, montmorillonite, and vermiculite.	Anolyte: synthetic wastewater; Inoculum: anaerobic digester sludge.	Batch mode.	MFC with the membrane prepared using red soil, montmorillonite and vermiculite: 995.73 ± 49.3 mW m^−3^.	[26]
Single-chamber air-cathode MFC; Anode: carbon veil sheet; Cathode: AC/PTFE mixture pressed on a stainless steel 316 mesh.	Ceramic separator containing 5.75 vol.% of iron oxide (sintered at 1100 °C).	Anolyte: urine; Inoculum: partially hydrolysed urine and effluent from running MFCs.	Continuous-flow mode.	Max. power output: 1.045 mW.	[79]
Single-chamber air-cathode MFC; Anode: carbon felt; Cathode: carbon felt.	Red soil ceramic membrane modified using montmorillonite, spray-coated with Nafion solution.	Anolyte: synthetic solution containing sodium acetate.	Batch mode (3 days per cycle).	84.3 mW m^−3^.	[78]
Two-chamber MFC; Anode: graphite felt; Cathode: graphite felt.	5% goethite in natural clay (G-5).	Anolyte: synthetic wastewater with sucrose; Inoculum: anaerobic mixed sludge; Catholyte: water.	Batch mode; 27 ± 5 °C.	The G-5 membrane: 112.81 ± 8.74 mW m^−2,^ which is slightly higher than 106.95 ± 5.52 mW m^−2^ produced by Nafion-117.	[33]
Air-cathode cylindrical MFC; Anode: plain carbon fibre veil; Cathode: hot-pressed activated carbon.	Mixture of plastic clay and chamotte: named brown, red, and white.	Anolyte: human urine; Inoculum: anaerobic sewage sludge.	Batch mode; White and red ceramics are more porous and have smaller pores compared to brown ceramic.	White ceramic-based with brown spots: 71.8 W m^−3^; White ceramic-based with red spots: 71.5 W m^−3^.	[77]
Air-cathode MFC: terracotta bottom sealed cylinders; Cathode: activated carbon.	Terracotta separators modified with ionic liquid (IL), [EMIM][Tf2N].	Anolyte: activated sewage sludge with sodium acetate.	Batch mode.	Terracotta separators modified with ionic liquid, [EMIM][Tf2N], achieved up to 86.5% more power output compared to IL-free MFCs from 229.78 µW to 428.65 µW.	[75]
Air-cathode, single-chamber MFC; Anode: carbon veil; Cathode: conductive graphite paint.	pyrophyllite; earthenware; mullite; alumina.	Inoculum: anaerobic activated sludge; Anolyte: fresh human urine.	Continuous flow; Flow rate: 0.35 L d^−1^.	6.93 W m^−3^ (pyrophyllite); 6.85 W m^−3^ (earthenware); 4.98 W m^−3^ (mullite); 2.60 W m^−3^ (alumina).	[76]
Two-chamber MFC; Anode: stainless steel mesh; Cathode: carbon felt.	Red soil separator—rich in aluminum and silica; Black soil—rich in Ca, Fe, and Mg.	Anolyte: synthetic wastewater with sodium acetate; Inoculum: anaerobic mixed sludge; Catholyte: tp water (aerated).	Batch mode; 33–37 °C.	MFC with Red soil separator: 1.49 W m^−3^ and 51.65 mW m^−2^ anode area; MFC with Black soil separator: 1.12 W m^−3^ and 31.20 mW m^−2^ anode area.	[74]

AC, activated carbon; [EMIM][Tf2N], 1-ethyl-3-methylimidazolium bis(trifluoromethylsulfonyl)imide, PTFE, polytetrafluoroethylene; PVDF, polyvinylidene fluoride; PVDF-OH, alkaline-treated polyvinylidene fluoride; PVDF/BS, rhamnolipid-treated polyvinylidene fluoride; WWTP, wastewater treatment plant.
